# Pretreatment with Relaxin Does Not Restore NO-Mediated Modulation of Calcium Signal in Coronary Endothelial Cells Isolated from Spontaneously Hypertensive Rats

**DOI:** 10.3390/molecules20069524

**Published:** 2015-05-26

**Authors:** Silvia Nistri, Lorenzo Di Cesare Mannelli, Carla Ghelardini, Matteo Zanardelli, Daniele Bani, Paola Failli

**Affiliations:** 1 Departments of Clinical & Experimental Medicine, Research Unit of Histology & Embryology University of Florence, Viale G. Pieraccini 6, 50139 Florence, Italy; E-Mails: silvia.nistri@unifi.it (S.N.); daniele.bani@unifi.it (D.B.); 2 NEUROFARBA, Section of Pharmacology & Toxicology, University of Florence, Viale G. Pieraccini 6, 50139 Florence, Italy; E-Mails: lorenzo.mannelli@unifi.it (L.D.C.M.); carla.ghelardini@unifi.it (C.G.); mattezana.mz@gmail.com (M.Z.)

**Keywords:** angiotensin II, cGMP-dependent protein kinase I, cardiovascular diseases, NG-nitro-l-arginine methylester, normotensive Wistar Kyoto rats, *S*-nitroso-*N*-acetylpenicillamine, α-thrombin, W1400

## Abstract

We demonstrated that in coronary endothelial cells (RCEs) from normotensive Wistar Kyoto rats (WKY), the hormone relaxin (RLX) increases NO production and reduces calcium transients by a NO-related mechanism. Since an impairment of the NO pathway has been described in spontaneously hypertensive rats (SHR), the present study was aimed at exploring RLX effects on RCEs from SHR, hypothesizing that RLX could restore calcium responsiveness to NO. RCEs were isolated from WKY and SHR. Calcium transients were evaluated by image analysis after the administration of angiotensin II or α-thrombin. Angiotensin II (1 µM) caused a prompt rise of [Ca^2+^]i in WKY and SHR RCEs and a rapid decrease, being the decay time higher in SHR than in WKY. NOS inhibition increased calcium transient in WKY, but not in SHR RCEs. Whereas RLX pretreatment (24 h, 60 ng/mL) was ineffective in SHR, it strongly reduced calcium transient in WKY in a NO-dependent way. A similar behavior was measured using 30 U/mL α-thrombin. The current study offers evidence that RLX cannot restore NO responsiveness in SHR, suggesting an accurate selection of patients eligible for RLX treatment of cardiovascular diseases.

## 1. Introduction

In the vascular system, the nitric oxide (NO) pathway mediates vasorelaxation and platelet anti-aggregation and protects from ischemic disorders [1]. NO, physiologically produced by different nitric oxide synthase isoforms (eNOS, nNOS and iNOS), can activate soluble guanylyl cyclase (sGC) to produce cyclic guanosine monophosphate (cGMP), which in turn activates the cGMP-dependent protein kinase (cGK-I), modulating ion channels, phosphodiesterases and calcium pumps [2]. In this context, convincing evidences exist in the literature that the hormone relaxin (RLX) can promote coronary and systemic vasodilatation by increasing NO bioavailability and NOS enzyme expression [3], thereby reducing hypertension and protecting the heart against ischemia/reperfusion-induced injury [4,5]. Thus, administration of recombinant human H2 RLX, or serelaxin, has been proposed as a potential therapeutic strategy for hypertension and heart ischemia [6].

The spontaneously hypertensive rat (SHR) is an animal model used for the study of hypertension, hypertensive heart disease, cardiac remodeling and hypertrophy. In this model, alterations in Ca^2+^ handling have been described at very early stages of the disease, even before the appearance of cardiac remodeling [7]. Many different factors are involved in this spontaneous, age-dependent pathological condition, including an impairment of the NO pathway. In particular: (i) mRNA expression of cGMP-dependent protein kinase I (cGKI) was found reduced in aortic rings of 6 week-old SHR [8]; (ii) decreased cGK activity was detected in ventricular and atrial tissue of aged SHR [9] and other forms of hypertensive animals [10] and (iii) and, according to our research data, the cGKI expression is reduced in cardiomyocytes and coronary endothelial cells (RCEs) of 12 week old SHR [11,12]. Of note, cGKI is a major regulator of intracellular calcium homeostasis and its over-expression was found to restore NO-mediated calcium regulation in RCEs and aortic smooth muscle cells isolated from SHR [12,13]. 

Along this line of thought, the administration of RLX to in female non-pregnant SHR was reported to cause a sustained decrease in blood pressure [14] and to substantially blunt the vascular response to vasoconstrictors in mesenteric vasculature but not in portal vein [15]. Besides these acute functional effects, RLX was also capable of reverting arterial adverse remodeling and decreased compliance in elderly SHR [16].

The cellular mechanisms underlying these vascular effects of RLX are not fully understood but represent a topic worthy of investigation because of their obvious medical interest. In this context, previous studies performed by our team on RCEs from Wistar Kyoto (WKY) rats, the normotensive counterpart of SHR, have demonstrated that RLX increases NO production by up-regulating NOS expression and decreases vasoconstrictor-induced intracellular calcium concentration ([Ca^2+^]i) rise by a NO-related mechanism [17]. Therefore, it was reasonable to assume that a similar mechanisms may also be operating in SHR. 

In the present study we aimed at exploring the effects of RLX on RCEs isolated from SHR, based on the working hypothesis that RLX may restore [Ca^2+^]i responsiveness to NO. According to our previous study on WKY and SHR, we used angiotensin II (AT-II) and α-thrombin (THR) to induce [Ca^2+^]i increase in RCEs, since these cells have been shown to express AT-II and THR receptors and to respond to exogenous AT-II and THR by modulation of NO-dependent [Ca^2+^]i increase [12,18,19]. Preliminary data were presented at the Fourth International Conference on “Relaxin and related peptides” [20].

## 2. Results

### 2.1. Intracellular Ca^2+^ Control Conditions

**Figure 1:**
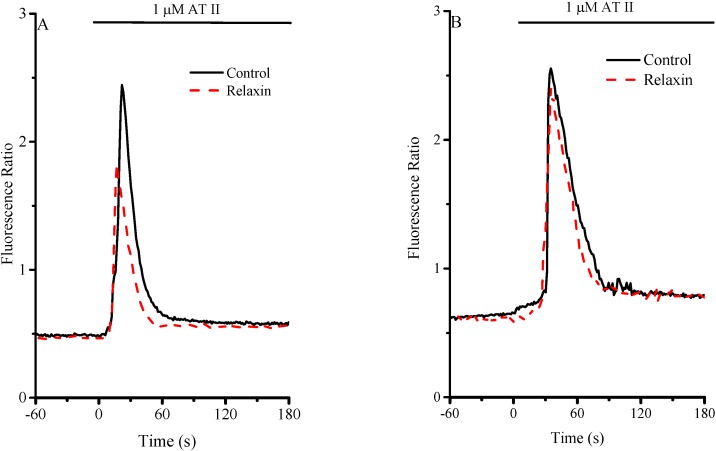
Evaluation of [Ca^2+^]i. in RCE isolated from WKY and SHR rats. (**A**) Representative tracings of [Ca^2+^]i-associated fluorescence in Fura-2 RCEs from WKY rats in control and RLX-pretreated cultures upon challenge with AT-II; (**B**) Representative tracings of [Ca^2+^]i-associated fluorescence in Fura 2-loaded RCEs from SHR in control and RLX-pretreated cultures upon challenge with AT-II. AT-II (1 μM) was added at the arrow (time = 0) and maintained throughout the experiment, as indicated. Cells were incubated for 24 h in serum-free medium in the absence (controls) or presence of 60 ng/mL RLX.

At baseline, [Ca^2+^]i, evaluated by Fura 2 fluorescence, was 112.5 ± 2.76 nM in RCEs isolated from WKY and slightly higher in those from SHR (144.1 ± 7.43 nM). Stimulation of RCEs with 1 µM AT-II, caused a prompt rise of [Ca^2+^]i in both WKY and SHR strains (Figure 1). 

**Figure 2:**
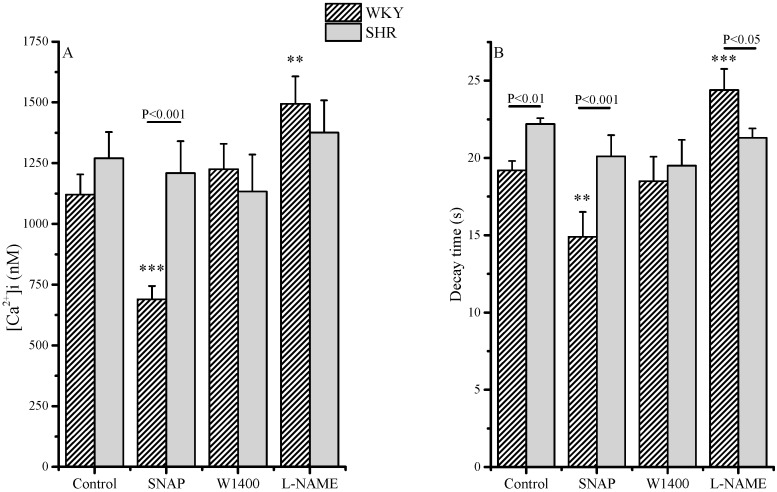
Evaluation of [Ca^2+^]i in RCE isolated from WKY and SHR rats. [Ca^2+^]i increase (maximal value at the peak, (**A**) and [Ca^2+^]i transient decay time (**B**) in WKY and SHR RCEs. SNAP (100 µM), L-NAME (10 µM) or W1400 (1 µM) were incubated 20 min before the administration of AT-II (1 µM). Values are the mean (±S.E.M.) of data from three separate RCE cell isolations, where at least 20–24 cells for each type of protocol were separately analyzed. Segments on the top of bars indicate significance between WKY and SHR; stars indicate significance between treatment and control, same strain: *** *p* < 0.001; ** *p* < 0.01.

In the SHR cells, the maximum [Ca^2+^]i increase was slightly, albeit not significantly higher than in those from WKY (Figure 2A). Calcium signals decreased rapidly in WKY RCEs with a decay time of 19.2 ± 0.61 s, whereas in SHR cells, the decay time was significantly higher (Figure 2B). A 10 min. incubation with the NO-donor SNAP significantly decreased maximum [Ca^2+^]i increase and decay time in the WKY cells, whereas it was ineffective in the SHR RCEs (Figure 2). Moreover, 20 min. preincubation with the non-selective NOS inhibitor L-NAME significantly increased maximum [Ca^2+^]i increase and decay time in the WKY, but not in the SHR cells. Inhibition of NOS II with W1400 had no effect on calcium transient in both strains. 

The data depicted in Figure 2 were also reported in Table 1 as differences (Delta) between the calcium transient parameters in control (basal) and incubated cells with SNAP, W1400 or L-NAME. 

**Table 1:** Differences among control and incubated cells with the specified molecules in basal control in RCEs isolated from WKY and SHR after AT-II activation of calcium transient.

	**Delta [Ca^2+^]i (nM)**	
	WKY	SHR
Control (basal)	0	0
SNAP	−430	−61
W1400	+150	−137
L-NAME	+374	+106
RLX-pretreated	−223	+3
	**Delta Decay Time (s)**	
	WKY	SHR
Control (basal)	0	0
SNAP	−4.3	−2.1
W1400	−0.7	−2.7
L-NAME	+5.2	−0.9
RLX-pretreated	−5	−1.9

The effect of RLX pretreatment is also reported. In WKY RCEs, maximal calcium values and decay times obtained were strongly reduced by SNAP and RLX, whereas L-NAME increased both parameters. W1400 only marginally influenced calcium transients, suggesting that under basal conditions the NO production was mainly dependent on the activation of a constitutive endothelial NOS. On the contrary, in SHR RCEs SNAP, NOS inhibition or RLX pretreatment were ineffective. 

### 2.2. Intracellular [Ca^2+^]: RLX Effects

**Figure 3:**
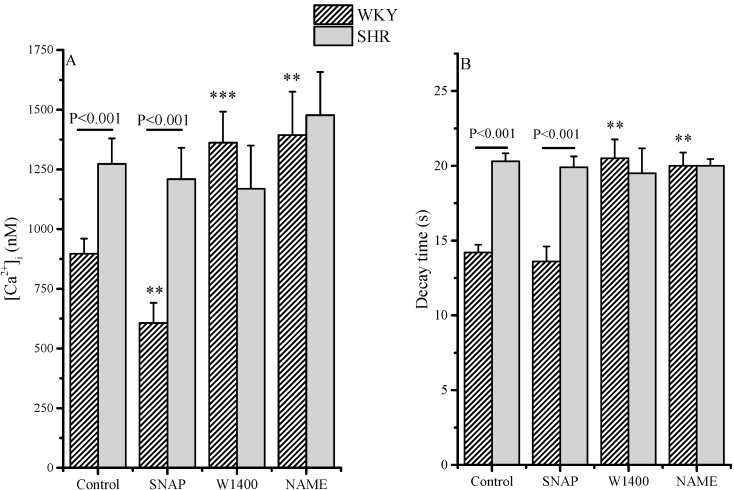
Evaluation of [Ca^2+^]i in RCE isolated from WKY and SHR rats pretreated with RLX. [Ca^2+^]i increase (maximal value at the peak, (**A**) and [Ca^2+^]i transient decay time (**B**) in WKY and SHR RCEs. Cells were pretreated with RLX (60 ng/mL) for 24 h; SNAP (100 µM), L-NAME (10 µM) or W1400 (1 µM) were incubated 20 min. before the administration of AT-II (1 µM). Values are the mean (±S.E.M.) of data from three separate RCE cell isolations, where at least 20–24 cells for each type of protocols were analyzed separately. Segments on the top of bars indicate significance between WKY and SHR; stars indicate significance between treatment and control, same strain: *** *p* < 0.001; ** *p* < 0.01.

In WKY RCEs, a 24-h incubation with RLX decreased both peak and decay time of the agonist-induced [Ca^2+^]i transient (Figure 1A, Figure 3): the effect of RLX preincubation was potentiated by a 10-min. incubation with SNAP. 

A 20 min incubation with either the nonspecific NOS inhibitor L-NAME or the selective NOS II inhibitor 1400W modified the kinetics of AT-II induced [Ca^2+^]i transients in RLX-pretreated WKY RCEs: as shown, both inhibitors increased the maximum and the decay time of the calcium transient. A 24 h incubation with RLX in SHR cells was ineffective in reducing the calcium transients, as evaluated by maximum values at the peak and decay times. Again, in SHR cells, the short incubation with SNAP or with both NOS inhibitors did not modify calcium transients in the RLX-pretreated cells.

The effect of RLX pretreatment on the calcium transient induced by AT-II is reported in Table 2 as delta. In WKY, SNAP was still effective in reducing the maximal calcium value, while it influenced to a minimal extent decay time in RLX-pretreated cells. W1400 and L-NAME strongly increased calcium transient as indicated by the delta differences among in RLX-pretreated control and NOS inhibitors. The direct comparison of the alcium transient value of untreated cells shows that RLX effectively reduced the delta calcium and decay time value. On the contrary, in SHR RCEs RLX was ineffective and nor SNAP or NOS inhibition modified calcium transients. 

**Table 2:** Differences among control and incubated cells with the specified molecules in RLX-pretreated RCEs isolated from WKY and SHR after AT-II activation of calcium transient.

	**Delta [Ca^2+^]i (nM)**	
	WKY	SHR
Control (RLX-pretreated)	0	0
SNAP	−281	−64
W1400	+465	−104
L-NAME	+495	+204
Control (basal)	+223	−3
	**Delta Decay Time (s)**	
	WKY	SHR
Control (RLX-pretreated)	0	0
SNAP	−0.6	−0.4
W1400	+6.3	−0.8
L-NAME	+5.8	−0.3
Control (basal)	+5	+1.9

### 2.3. Effect of RLX on α-Thrombin-Induced Calcium Transient 

As already described [19], 30 U/mL THR induced a rise of [Ca^2+^]i in RCEs of both strains (Table 3). RLX pretreatment significantly reduced both parameters of calcium transient (*i.e.*, maximal calcium peak and decay time) in WKY cells. Again, in SHR RCEs THR-induced calcium transients were not modified by RLX pretreatment (Table 3).

**Table 3:** Effect of RLX-pretreatment (60 ng/mL) for 24 h) on 30 U/mL α-thrombin-induced calcium transient in coronary endothelial cells isolated from WKY and SHR rats.

Control (basal)	WKY	SHR
[Ca^2+^]i (Maximal value)	1820 ± 185.3 nM	1901 ± 178.6 nM
Decay time	18.1 ± 0.83 s	19.0 ± 0.64 s
**24h RLX-Pretreated**		
[Ca^2+^]i (Maximal value)	1168 ± 146.4 nM **, §§	1841 ± 206.1 nM
Decay time	13.4 ± 1.19 s ***, §§	17.5 ± 2.03 s

** *p* < 0.01 *vs.* WKY control, *** *p* < 0.001 *vs.* WKY control; §§ *p* < 0.01 *vs.* SHR RLX-pretreated.

### 2.4. NO Production in WKY and SHR Endothelial Cells Pretreated with Relaxin

Accordingly to data already published [17], RLX increased NO production to 161% ± 17.2% (*n* = 3) in WKY and to 151% ± 21.3% (*n* = 3) in SHR RCEs as compared to controls (100%).

## 3. Discussion

The present data confirm that [Ca^2+^]i in RCEs isolated from normotensive WKY rats is strongly modulated by the NO pathway. In particular, while a NO donor decreases the calcium transient induced by AT-II, an aspecific inhibitor of NOS (L-NAME) can increase it. Moreover, the hormone RLX, known for its vasodilatory properties [5], reduces calcium transients in a NO-dependent mode related to NOS II [17]. These data validate the important role of NO in endothelial cells as a modulator of calcium signals in normotensive rats and suggest that NO can act in an autocrine manner in RCEs [12,17]. A remarkably different behavior is observed in SHR RCEs. In fact, in these cells, NO is unable to modulate [Ca^2+^]i and this ineffectiveness is maintained after treatment with RLX, suggesting that a downstream step of the NO pathway is altered. Similarly, using THR as calcium agonist, RLX-pretreatment strongly reduces calcium transients in WKY, whereas it is ineffective in SHR RCEs. Since RLX-pretreatment significantly lowers the calcium transients induced by two different agonists (*i.e.*, AT-II and THR) only in WKY, the disfunctionality in SHR cells should lie in the NO pathway instead of the specific receptor signal. In this context, we have previously reported that SHR RCEs show low/absent expression of cGKI enzyme [12]. Moreover, a similar reduction of cGKI has been described in cardiomyocytes [11] and aortic smooth muscle cells [13]. The present data demonstrate that RLX is unable to restore NO responsiveness in SHR RCEs and appears to be partially in disagreement with previous *in vivo* studies in which RLX reduced blood pressure [14], cardiac and renal fibrosis [21] in SHR. This discrepancy may depend on many reasons. Multiple signal transduction pathways are activated in response to relaxin [6]. Indeed, RLX receptors are coupled with different G proteins, including the Gs cyclic AMP-stimulating protein [22]. Therefore *in vivo* effects of RLX in SHR could be mediated by other pathways. Of note, a direct NO-mediated relaxant effect of RLX on smooth musculature has been consistently described in other target organs, including smooth muscle cells [23], the uterus [24,25] and the gastrointestinal tract [26].

The current study evidences that RLX cannot restore NO responsiveness in SHR RCEs and underlines the importance of the NO/sGK/cGKI pathway in controlling the [Ca^2+^]i dynamics presiding to the regulation of vascular tone.

A genome-wide association study found that common genetic polymorphisms in human cGKI-1 gene (*PRKG1*) are significantly associated with enhanced diastolic blood pressure in response to an acute salt load in patients with hypertension [27]. Chronically elevated blood pressure increases left ventricular (LV) pressure, enhances LV radial systolic performance and leads to LV hypertrophy. Recently, LV systolic radial deformation (strain) has been associated with common polymorphisms in *PRKG1* [28]. In particular, LV radial systolic deformation is significantly higher in patients carrying *PRKG1* homozygote polymorphism than in heterozygotes and noncarriers. This knowledge may have clinical implications, as it suggests that NO-modulating drugs (including RLX) used for cardiovascular diseases might be low effective or ineffective in these patients. Even if further studies must be undertaken to elucidate how the genetic variants of *PRKG1* might influence cardiovascular diseases, in homozygote *PRKG1* polymorphisms carriers, RLX could be ineffective in the treatment of hypertension and hypertensive heart diseases, suggesting an accurate evaluation of RLX effect in clinical setting.

## 4. Experimental Section

### 4.1. Chemicals

Highly purified porcine RLX (2500–3000 U/mg) was a generous gift from O. D. Sherwood. RLX was used at a concentration of 60 ng/mL, which is in the range found effective in inducing coronary vasodilatation in rat hearts [29]. Media, sera, and reagents for cell culture were from Sigma-Aldrich (Milan, Italy) and Gibco Life Technologies (Milan, Italy). Cell culture plastic ware was purchased from Costar (Corning Costar Co., Costar Italia, Milan, Italy). Fura 2-AM and Pluronic F127 were from Molecular Probes Life Technologies (Milan, Italy). The selective NOS II inhibitor 1400W [30] was from Alexis Biochemicals (Enzo Life Sciences, New York, USA) and the NO-donor *S*-nitroso-*N*-acetylpenicillamine (SNAP) from Tocris (Bristol, UK). THR was from Roche Life Sciences, NG-nitro-l-arginine methylester (L-NAME) and angiotensin II (AT II) were from from Sigma-Aldrich as were the other chemicals used.

### 4.2. Isolation and Culture of Rat Coronary Endothelial (RCE) Cells

RCEs were isolated from the heart of 3–4-month old male Wistar Kyoto and aged matched SHR rats, as described previously [19]. Rats (Charles River, Lecco, Italy) were housed in the Centro per la Stabulazione degli Animali da Laboratorio (Ce.S.A.L., University of Florence), maintained for at least one week after their arrival in a 12 h dark-light cycle with pellet food and water *ad libitum*. Formal approval to conduct the experiments described was obtained from the Animal Subjects Review Board of the University of Florence. The ethics policy of the University of Florence complies with the Guide for the Care and Use of Laboratory Animals of the U.S. National Institutes of Health (NIH Publication No. 85-23, revised 1996; University of Florence Assurance No. A5278-01). 

Briefly, after enzymatic digestion of the heart, the suspension was centrifuged and the pellet was stirred for 30 min at 37 °C in the presence of 10 mg/50 mL trypsin. The recovered pellet was resuspended in 15 mL of culture medium (see below), and plated. After 4 h, cells were washed twice and grown until confluence (5–6 days) in M199 containing 10% fetal calf serum (FCS), 10% newborn calf serum, 250 U/mL penicillin G, 0.625 µg/mL amphotericin, and 250 µg/mL streptomycin. Isolated RCEs were cytocharacterized as previously reported [19,31]. Cells immunoreactive for endothelial markers ranged between 96% and 98%. For all experiments, cells were used at the first passage. Stimulation of RCEs with RLX was carried out in M199 medium without phenol red.

### 4.3. Determination of Intracellular Ca^2+^

Intracellular cytosolic Ca^2+^ ([Ca^2+^]i) was evaluated with Fura-2 by microscopic image analysis as described previously [12,19]. Briefly, cells were grown on round cover slips to subconfluence and then incubated for 24 h in serum-free medium in the absence (controls) or presence of RLX (60 ng/mL). Cells were loaded with the Ca^2+^-sensitive fluorescent probe Fura 2-AM (4 µmol/L) and Pluronic F (0.02%) for 45 min at room temperature in HEPES-bicarbonate buffer containing (mM): NaCl 140, KCl 2.9, NaH_2_PO_4_ 0.5, NaHCO_3_ 12, MgCl_2_ 0.9, HEPES 10, CaCl_2_ 1, glucose 10, adjusting pH to 7.4 with NaOH 1 N.

Coverslips were washed and mounted in the stage of an epifluorescence inverted microscopy in a perfusion chamber warmed at 35 °C. [Ca^2+^]i was measured dynamically by monitoring Fura-2 fluorescence ratio. Consecutive images, obtained with excitation wavelengths of 340 and 380 nm and an emission wavelength of 510 nm, were recorded with a charge coupled device (CCD) video camera and digitized by an analogical/digital converter (resolution: 256 × 256 pixels, time interval between subsequent images: 800 ms). Image ratios (340/380 nm) were obtained every 3 s on a pixel-to-pixel basis after subtraction of the background. 

After measurements of [Ca^2+^]i in basal conditions, 1 µM AT II or 30 U/mL of THR were added directly into the perfusion chamber, and the time course of [Ca^2+^]i increase induced by agonists was analyzed for at least 15 min as already described [12,19]. A noise/signal ratio of 0.5 of 340/380 nm fluorescence was considered as the lowest detectable limit for [Ca^2+^]i transient. In some experiments, 100 μM SNAP, the broad-spectrum NOS inhibitor L-NAME (10 µM) or the specific NOS II inhibitor 1400W (1 µM) were added to the perfusion chamber 20 min before the addition of the agonist. Calibration curves were performed using a Kd of 214 nM for Fura-2.

The experimental data were exported as ASCII file format and graphically elaborated with MicroCal Origin 9 software (OriginLab, Northhampton, MA, USA). The decay time of calcium transient was calculated as reported previously [19] and expressed as the time (s) needed for fluorescence ratio to reach 36.79% of the maximal value. The reported values are the mean (±SE) of data from three separate RCE cell isolations, where at least 20–24 cells for each type of protocols were analyzed separately.

NO production was evaluated in the medium of control and 24h RLX pretreated cells by measuring nitrite production with Griess reagent as already described [17].

### 4.4. Calculations and Statistical Analysis

Statistical comparisons were performed by using one-way ANOVA test followed by Student-Newman-Keuls multiple comparison test. A *p value* ≤ 0.05 was considered as the low level of significance.
